# Vision-Language Model-Based Local Interpretable Model-Agnostic Explanations Analysis for Explainable In-Vehicle Controller Area Network Intrusion Detection

**DOI:** 10.3390/s25103020

**Published:** 2025-05-10

**Authors:** Jaeseung Lee, Jehyeok Rew

**Affiliations:** 1 School of Electrical Engineering, Korea University, Seoul 02841, Republic of Korea; jason2133@korea.ac.kr; 2 Department of Data Science, Duksung Women’s University, Seoul 01370, Republic of Korea

**Keywords:** vision-language model, controller area network, anomaly detection, vehicle intrusion detection, explainable artificial intelligence, local interpretable model-agnostic explanation

## Abstract

The Controller Area Network (CAN) facilitates efficient communication among vehicle components. While it ensures fast and reliable data transmission, its lightweight design makes it susceptible to data manipulation in the absence of security layers. To address these vulnerabilities, machine learning (ML)-based intrusion detection systems (IDS) have been developed and shown to be effective in identifying anomalous CAN traffic. However, these models often function as black boxes, offering limited transparency into their decision-making processes, which hinders trust in safety-critical environments. To overcome these limitations, this paper proposes a novel method that combines Local Interpretable Model-agnostic Explanations (LIME) with a vision-language model (VLM) to generate detailed textual interpretations of an ML-based CAN IDS. This integration mitigates the challenges of visual-only explanations in traditional XAI and enhances the intuitiveness of IDS outputs. By leveraging the multimodal reasoning capabilities of VLMs, the proposed method bridges the gap between visual and textual interpretability. The method supports both global and local explanations by analyzing feature importance with LIME and translating results into human-readable narratives via VLM. Experiments using a publicly available CAN intrusion detection dataset demonstrate that the proposed method provides coherent, text-based explanations, thereby improving interpretability and end-user trust.

## 1. Introduction

The Controller Area Network (CAN) is a widely adopted in-vehicle communication protocol that enables data exchange among electronic control units (ECUs) [1]. It plays a key role in managing essential vehicle operations such as engine control, braking systems, and airbag deployment, which are directly associated with driving performance and safety. By facilitating real-time communication, the CAN supports timely and synchronized coordination among vehicle components [2]. However, as the CAN protocol was initially designed with a focus on efficiency rather than security, its structure is susceptible to potential exploitation by unauthorized entities [3]. In the absence of an intrusion detection system (IDS), these vulnerabilities can be exploited, posing significant risks to the safe and reliable operation of the vehicle [4].

Intrusion detection for CAN is important for enhancing vehicle security and ensuring driver safety [5]. Unauthorized access to CAN may allow attackers to manipulate in-vehicle data, potentially compromising critical control systems and resulting in unsafe operational behavior. For example, an attacker could manipulate brake signals to interfere with the braking function or tamper with accelerator signals, potentially causing unintended acceleration. These security threats can be addressed by deploying IDS techniques that monitor CAN communication patterns and detect anomalous behaviors that may signal malicious activity [6]. In addition, a timely IDS response can significantly reduce the risk of security breaches and enhance the overall safety of vehicle systems. Furthermore, context-aware defense mechanisms can be automatically activated based on the type of detected attack, thereby reinforcing the vehicle’s overall security architecture.

Earlier studies on CAN-based IDS primarily employed statistical methods to detect anomalies in communication patterns [5,7,8]. Recently, machine learning (ML)-based IDS methods have shown improved detection accuracy and generalization capabilities compared to traditional methods [1,9,10]. However, despite their high detection, these ML-based IDS models often exhibit complex internal structures. Such complexity limits the interpretability of the model, making it challenging to attribute specific input features to intrusion detection decisions within the CAN. As a result, they are frequently referred to as a ‘black box’ model. Furthermore, ML models are optimized for processing high-dimensional data, which presents challenges for visualization and intuitive understanding [11]. These aspects make it difficult to identify the root cause of errors when the model makes incorrect predictions, thereby limiting opportunities for model refinement. To address these limitations, it is essential to analyze the reasoning behind the model’s predictions and enhance both the accuracy and reliability of the IDS through interpretability-focused methodologies.

Recently, explainable artificial intelligence (XAI) has garnered significant attention for its role in improving the transparency and reliability of ML models [12]. XAI encompasses techniques that interpret the decision-making process of artificial intelligence models and present them in a form that is understandable to humans. In particular, XAI plays a vital role in mitigating the opacity of complex black-box models. This enhances the trustworthiness and accountability of AI-driven decision-making systems [13]. Various studies have been conducted to enhance the explainability of ML models, with well-known post hoc XAI techniques including Local Interpretable Model-agnostic Explanations (LIME) [14], Shapley Additive Explanations (SHAP) [15], and Partial Dependence Plots (PDP) [16].

However, most existing XAI techniques rely on graphical visualizations, which require users to manually interpret the results to understand the model’s predictions [17]. Given that users typically summarize these outputs in text, delivering XAI explanations in textual form can enhance both interpretability and usability [18]. In addition, to effectively interpret an ML model using XAI techniques, it is important to consider both global and local perspectives [19]. The global view analyzes the overall decision patterns and key features learned by the model, while the local view focuses on explaining individual predictions for specific inputs. Combining these perspectives enables users to understand both the general behavior of the model and the reasoning behind specific outcomes, thereby improving interpretability and trust.

Driven by recent advances in artificial intelligence, vision-language models (VLMs) have attracted growing attention [20]. These models are trained on large-scale datasets containing paired image and text data, allowing them to transcend basic image analysis and achieve a comprehensive understanding by integrating visual and linguistic information. VLMs are well suited for extracting semantic information from images, generating descriptive text, and analyzing the relationships between visual and textual data [21]. In addition to these capabilities, VLMs exhibit strong reasoning abilities, enabling them not only to describe visual content but also to perform complex tasks such as explaining decision pathways and interpreting final prediction results. This reasoning capacity makes VLMs particularly valuable for tasks requiring both interpretability and analytical insight. In particular, VLMs can analyze feature importance rankings by identifying which input variables consistently contribute to specific types of intrusions. For example, they can highlight that a particular data byte is identified as a key feature for detecting Fuzzy attacks based on its consistently high importance across multiple instances. Furthermore, the VLM can explain individual predictions by mapping specific feature values to the corresponding predicted class, thereby providing a clear rationale for why a given CAN message is classified as an intrusion.

The advantages of VLMs become more evident in intrusion detection tasks when compared to traditional ML models. Conventional models primarily focus on improving detection accuracy and identifying the presence of intrusions [22,23]. However, they lack the ability to provide detailed and interpretable explanations for their decisions. This limitation can be a significant drawback in CAN intrusion detection, where understanding the underlying causes of intrusions is essential for enabling proactive and effective responses. In contrast, VLMs, especially when integrated with XAI techniques, provide more precise and comprehensive insights into the specific input factors that contribute to each detected intrusion. This enhanced interpretability not only facilitates a deeper understanding of the detection results but also supports the development of more effective and timely countermeasures.

In this paper, we propose an explainable intrusion detection method for in-vehicle CAN by integrating LIME analysis with a VLM. An ML-based intrusion detection model is initially developed using LightGBM [24]. Then, the VLM is employed to assess the global impact of each input feature on the model’s predictions across the entire dataset. The VLM generates textual descriptions corresponding to the visualized global feature importance. Furthermore, the global feature importance extracted by the VLM is utilized as auxiliary knowledge in conducting LIME analysis. The VLM also produces textual explanations for individual predictions, effectively bridging global and local interpretability within a unified language-based method. To validate the effectiveness of the proposed method, we utilize a publicly available CAN intrusion detection dataset. In addition, through experiments using various prompt designs, we demonstrate the explainability and interpretive capabilities of the VLM-based LIME analysis.

The main contributions of this paper are summarized as follows:

1. We propose a novel explainable intrusion detection method for in-vehicle CAN that integrates LIME analysis with a VLM, enabling both global and local interpretability.
2. We develop a structured prompt template for guiding a VLM in producing informative textual explanations, enabling more effective interpretation of intrusion detection outcomes.
3. The proposed method is validated using a publicly available CAN intrusion detection dataset, and its effectiveness is demonstrated through prompt-based experiments evaluating interpretability and explanation quality.

This paper is structured as follows: Section 2 discusses previous research related to intrusion detection in the CAN. Section 3 introduces the method proposed in this study. In Section 4, we describe the dataset used in our experiments and the experimental settings. Section 5 presents the experimental results of the proposed method and conducts an in-depth analysis. Finally, Section 6 concludes the paper by summarizing the findings and discussing future research directions.

## 2. Related Works

### 2.1. Intusion Detection for In-Vehicle CAN Based on Statistical Method

The traditional method of intrusion detection for in-vehicle CAN is to construct a model that considers the statistical characteristics of CAN data. For example, Hoppe et al. [7] proposed an intrusion detection method that analyzes the frequency and intervals of network messages to detect anomalies in the vehicle network. Their work is one of the earliest attempts to apply data-driven techniques to CAN security and laid the foundation for frequency-based anomaly detection in vehicle networks. Muter and Asaj [25] extended this approach by statistically modeling the frequency characteristics of CAN messages to distinguish between normal and abnormal traffic. They introduced a framework that systematically measures deviations in message occurrence, enabling the detection of both known and unknown attacks. Similarly, Miller and Valasek [8] investigated the temporal distribution of CAN messages and observed that during attacks, the message intervals exhibit abnormal fluctuations. Their findings highlighted the importance of time-based analysis in detecting replay and spoofing attacks, particularly in real driving environments.

However, these statistical models are limited in capturing complex nonlinear relationships due to their reliance on a small number of hyperparameters. As a result, they fail to effectively model the rapidly fluctuating variables in CAN, leading to reduced intrusion detection performance.

### 2.2. Intusion Detection for In-Vehicle CAN Based on Machine Learning Model

With recent advancements in artificial intelligence, research on ML-based intrusion detection models for in-vehicle CAN has gained significant attention. These models have demonstrated superior detection performance compared to traditional statistical methods.

Taylor et al. [9] applied supervised learning techniques to CAN data, showing that such algorithms can effectively classify normal and attack states in vehicle networks. Their study laid the foundation for applying the ML classifier to vehicle networks, demonstrating that even simple models could outperform rule-based detection methods in certain scenarios. Zhang et al. [26] introduced a network intrusion detection model based on a three-way decision-making approach integrated with random forest. By evaluating attribute importance and employing three decision rules to divide attributes, their model improves detection precision and recall in complex network environments. Wang et al. [10] proposed an optimized ML model combining k-nearest neighbor with a genetic algorithm to detect various types of network attacks. The use of a genetic algorithm enabled automatic hyperparameter tuning and feature selection, contributing to improved detection accuracy across multiple attack scenarios.

As research continues, ML-based intrusion detection models for CAN are evolving toward more sophisticated architectures, enabling more accurate and faster anomaly detection than traditional rule-based statistical methods.

### 2.3. Application of Explainable Artificial Intelligence in Intrusion Detection for In-Vehicle CAN

In the context of intrusion detection for in-vehicle CAN, not only the performance of detection models but also their ability to explain the rationale behind predictions has become increasingly important. As a result, recent research has actively explored the application of XAI techniques to enhance the interpretability of these models.

Lundberg et al. [27] developed an in-vehicle CAN intrusion detection model using a deep neural network and applied SHAP analysis to interpret feature importance in the classification process. By leveraging SHAP values, they identified key byte-level data fields contributing to the prediction outcomes, thereby enhancing the explainability of the detection mechanism. Ahmed et al. [28] constructed an extreme gradient boosting (XGBoost)-based detection model and employed SHAP to identify critical network message identifiers and data fields involved in the classification process. By interpreting feature contributions across various attack scenarios, their study demonstrated the practical applicability of XAI in enhancing situational awareness for security analysts. Hassan et al. [29] focused on achieving model explainability for intrusion detection in vehicular networks by applying LIME analysis. They demonstrated how LIME analysis could be used to interpret the predictions of ML models, providing insights into the features influencing the detection of anomalies in vehicular networks.

The integration of XAI techniques into in-vehicle CAN intrusion detection systems offers the dual advantage of improving model transparency and increasing user trust by revealing key factors influencing detection decisions.

### 2.4. Vision-Language Model

VLMs have emerged as powerful tools that integrate visual and textual data, enabling a more comprehensive understanding of complex information. Recent research has explored their applications in structured data understanding and decision support across various domains.

For instance, Li et al. [30] introduced StrucTexT, a framework designed for structured text understanding within visually rich documents. By employing a segment-token-aligned encoder and a novel pre-training strategy, StrucTexT effectively captures multimodal information across text, image, and layout, thereby enhancing the model’s ability to process complex document structures. Similarly, Luo et al. [31] proposed Bi-VLDoc, a bidirectional vision-language modeling approach tailored for visually rich document understanding. This model leverages a vision-language hybrid-attention mechanism to fully explore interactions between visual and textual modalities, resulting in improved performance on various document understanding benchmarks. Furthermore, Li et al. [32] proposed a document object contrastive learning (DoCo), a framework that enhances visual document understanding through contrastive learning. By aligning multimodal document object features with visual features from large VLMs, DoCo improves the comprehension of text-rich documents, addressing the fine-grained feature collapse issue in VLMs.

While significant progress has been made in VLM-based analysis of images and tabular data, research on employing VLMs to interpret the results of XAI techniques remains relatively scarce. There has been limited exploration of how VLMs can be used to generate textual explanations for in-vehicle CAN intrusion detection models by analyzing the relationship between global and local explanations in XAI. Structuring the overall characteristics of the intrusion detection model and illustrating the connections between individual predictions in a textual format can greatly enhance users’ comprehension. In this paper, we propose a method that utilizes VLMs to provide textual interpretations of LIME analysis results for in-vehicle CAN intrusion detection models from both global and local perspectives. Our method aims to streamline the interpretation process of XAI results, reducing the cognitive load and effort required for analysis while enabling users to gain a clearer understanding of the model’s behavior.

## 3. Proposed Method

**Figure 1:**
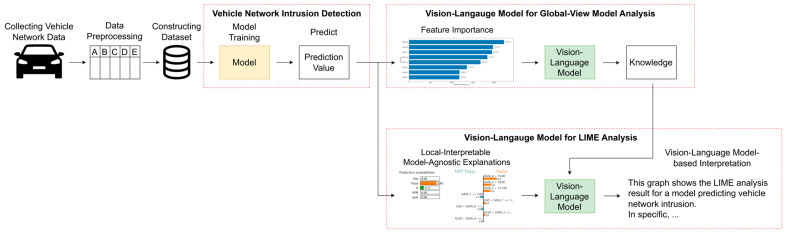
Overview of the proposed method.

This section explains the overall structure of the method proposed in this study. Figure 1 visually represents the overview of the proposed method.

### 3.1. In-Vehicle CAN Data Collection and Preprocessing

This study utilizes a CAN dataset collected during real vehicle operation, comprising both normal and attack messages. The attack types include denial-of-service (DoS), fuzzy, and spoofing attacks, making the dataset suitable for research on in-vehicle security [33].

All messages are labeled as normal or attack during data collection and are subsequently used as input for the intrusion detection model. In the data preprocessing stage, key features are extracted to capture the time-series characteristics of CAN messages and enhance model performance. First, missing values are removed to ensure data quality. Then, transmission intervals between CAN messages are analyzed, as attacks often cause abnormal timing patterns, including excessively repeated messages and irregular intervals. These timing anomalies serve as critical indicators for detecting intrusions. In addition, the occurrence frequency of specific CAN IDs is examined to differentiate between normal and attack behavior. Attackers tend to spoof or excessively transmit certain IDs, allowing the identification of abnormal message patterns. Furthermore, if the data field values of CAN messages deviate from the normal range or fluctuate abruptly without a consistent pattern, they can be considered a strong indication of an attack. By incorporating such features, the intrusion detection model is better equipped to identify anomalous messages.

Overall, the preprocessing procedure facilitates the extraction of informative input variables essential for detecting various types of attacks in CAN. By incorporating features that capture time-series characteristics, the proposed method enhances the robustness of the detection model and enables more precise differentiation between benign driving behavior and malicious activity.

### 3.2. In-Vehicle CAN Intrusion Detection Using Machine Learning

Based on the in-vehicle CAN dataset described in Section 3.1, an ML model is developed using LightGBM [24], a widely adopted framework recognized for its efficiency and predictive accuracy. To address the characteristics of the CAN intrusion detection task, a tree-based model was selected for its effectiveness in handling tabular, high-dimensional, and heterogeneous data typical of CAN messages. Unlike deep learning models, which require large datasets and significant computational resources to achieve high performance, LightGBM offers faster training, lower inference latency, and strong predictive capabilities even with moderate-sized datasets, making it suitable for real-time, resource-constrained in-vehicle environments. Furthermore, prior studies on in-vehicle network intrusion detection have also demonstrated that tree-based models, such as LightGBM and random forest, consistently achieve robust performance on tabular vehicle data [1,34,35]. As a framework based on gradient boosting decision trees (GBDT) [36], LightGBM further demonstrates scalability and maintains high predictive performance, even when applied to large datasets.

A notable advantage of LightGBM is its histogram-based learning algorithm, which discretizes continuous features into integer bins prior to training. This approach significantly reduces memory usage and accelerates computation, making it well-suited for high-frequency CAN message streams. In addition, LightGBM adopts a leaf-wise tree growth strategy that expands the leaf with the maximum loss reduction, rather than performing level-wise splits as in traditional GBDT models. This enables the construction of deeper, more complex trees with fewer nodes, improving the model’s ability to capture intricate relationships between normal and attack traffic patterns in CAN.

To enhance intrusion detection performance, we fine-tune key hyperparameters, including learning rate, tree depth, regularization strength, and sampling ratios, using grid search. This process is guided by validation loss, and early stopping is applied to prevent overfitting, ensuring a balanced trade-off between model complexity and generalization. As a result, the optimized LightGBM is able to effectively distinguish between normal and attack CAN messages while maintaining fast training and inference times suitable for in-vehicle deployment.

### 3.3. Vision-Language Model-Based Global Feature Importance Analysis for In-Vehicle CAN Intrusion Detection Model

**Figure 2:**
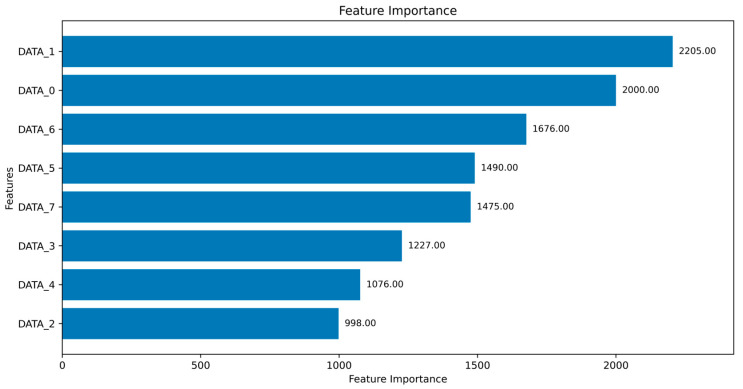
Visualization of global feature importance analysis.

A vision-language model (VLM) is employed to interpret global feature importance derived from the ML-based CAN intrusion detection model. After training the ML model, a global feature importance analysis is performed to measure how each input variable influences the model’s predictions [37]. As shown in Figure 2, the result is presented as a bar chart displaying the relative contribution of each feature. Global feature importance is typically assessed based on how frequently a feature is used to split decision nodes or how much it contributes to reducing prediction error during model training [38]. In this study, the 8-byte data field from each CAN message, denoted as DATA_0 through DATA_7, represents raw byte-level information that corresponds to sensor readings and system status flags. For example, Figure 2 shows that DATA_1 and DATA_0 have the highest importance scores, suggesting that the model heavily relies on these bytes when distinguishing between normal and intrusive CAN messages. The consistently high importance of DATA_1 may imply that it encodes a critical sensor value exhibiting distinctive patterns under attack conditions, thereby enabling the model to more effectively detect anomalies.

While a graphical representation of feature importance can provide valuable insights, it may not be sufficient for comprehensively understanding the model’s decision process [39]. Visualizations such as bar plots indicate which features contribute to predictions, but they do not explain why those features are important or how they interact. Moreover, the interpretation of such visualizations often depends on the user’s domain expertise, which may lead to inconsistent interpretations across users. Given these limitations and considering that users inherently rely on textual reasoning to interpret visual data, converting the feature importance graph into a descriptive textual explanation becomes essential. To bridge this gap between visual analysis and human interpretability, the VLM is employed to generate textual explanations based on feature importance visualizations. The VLM accepts a feature importance graph as input and generates descriptions explaining why the model emphasizes specific variables, how input variables are interrelated, and which features are critical for detecting different types of intrusions. By leveraging its ability to interpret visual data and generate text output, the VLM can automatically identify key input variables from the feature importance graph and explain their roles in the model’s decision-making process. This enables a clearer understanding of the model’s overall logic and the contribution of individual input variables from a global perspective.

The quality of the VLM’s output is highly dependent on the structure and phrasing of the input prompts [40]. Therefore, designing well-structured prompts is essential to ensure accurate and insightful explanations [41]. Table 1 presents the prompt template that is used to interpret global feature importance. The prompt is composed of three main components—role description, task specification, and interpretation guideline. The role description defines the identity and expertise of the model. This encourages the VLM to adopt a technical and analytical tone, appropriate for generating research-level interpretations. The task specification provides detailed contextual information, including the model’s purpose, the nature of the classification task, the meaning of the input variables, and the interpretation of the graph’s axes and numeric values. The name of the prediction model, indicated in curly braces, varies depending on the specific model employed. This helps the VLM understand the semantic content of the visual input. Lastly, the interpretation guideline outlines analytical objectives and constraints. In particular, the guideline explicitly instructs the model to avoid associating specific features with classes, emphasizing that the analysis is based on global feature importance. Furthermore, numbers are used to represent major analysis steps that require sequential reasoning, whereas dashes are utilized to indicate subsidiary details or writing guidelines within each step. This design choice is made to enhance clarity and maintain a hierarchical structure, thereby facilitating a more organized and effective interpretation process for the VLM. This ensures that the response of the VLM remains objective and comprehensive for reporting.

To further ensure the stability and consistency of the VLM outputs, the input prompts are standardized across all inference tasks. By maintaining consistent instruction structures, formatting styles, and contextual information, variability arising from prompt sensitivity is minimized. As a result, the generated textual explanations reliably capture the underlying feature importance distributions.

## 3.4. Vision-Language Model-Based LIME Analysis for Enhancing Explainability of In-Vehicle CAN Intrusion Detection Model

Analyzing global feature importance alone has limitations in fully explaining how the model makes predictions for individual data instances. While feature importance indicates how frequently the model utilizes specific variables across the entire dataset, it does not provide insights into the decision-making process for individual samples [42]. Therefore, we propose a method using LIME analysis to examine the model’s decision-making process for specific data samples and to identify the key input variables that play a significant role in each case.

LIME is a widely used technique for interpreting the prediction results of ML models, often referred to as a black-box model [14]. It operates by generating perturbed samples around an original instance and analyzing the model’s predictions on these samples to identify important input variables. This method allows for a quantitative assessment of how specific input variables influence the model’s predictions and provides an explanation for the reasoning behind the predictions. For example, when a model detects a specific CAN message as an intrusion, LIME can be used to determine whether this classification was driven by changes in specific attribute values or the repetitive occurrence of certain patterns.

**Figure 3:**
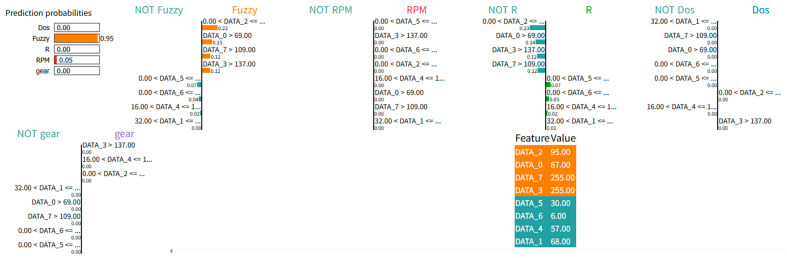
Visualization of LIME analysis.

However, LIME typically presents its results in visual formats such as bar charts or decision rule paths, as shown in Figure 3. These must be manually interpreted and translated into textual explanations by the user. For example, in Figure 3, features such as DATA_2 and DATA_0 appear frequently across multiple decision rule paths, reflecting their relevance to the model’s decision logic. This suggests that these features encode highly discriminative information that contributes to distinguishing the Fuzzy class from other intrusion types. To address this interpretability challenge, we utilize a VLM to automatically convert LIME visualization into structured textual explanations. This enables more accessible and human-readable interpretations of instance-level model behavior. Moreover, interpreting model behavior solely from a local perspective provides only a partial view of the model’s decision-making logic [19]. Global feature importance describes which variables are most frequently used across the entire dataset, while LIME-based local explanations reveal which features influenced the decision for a specific instance. To address this, we utilize the global feature importance results derived in Section 3.3 as prior knowledge to convert LIME analysis results into textual explanations, providing a more detailed interpretation of the model’s decision-making process. Table 2 presents the template of the prompt used to interpret LIME analysis results using the VLM.

The prompt is composed of four main components—role description, task specification, interpretation guideline, and contextual reference. The role description defines the VLM as a domain expert in ML and XAI, prompting it to adopt a technically precise tone. This is critical to ensure that the generated explanations meet the standards expected in reporting. The task specification introduces the necessary background, including the nature of the CAN intrusion detection task, the structure of the input data, and the interpretation of the LIME visualization. Similarly to Table 1, the name of the prediction model, the predicted class, the probability associated with the prediction, and the LIME analysis results are presented in curly braces. These elements vary depending on the prediction model used and the characteristics of the input data. This component enables the VLM to interpret the visual content meaningfully and tailor its explanation to the specific instance under analysis. The interpretation guideline defines the analytical scope and structure of the explanation. It instructs the model to assess the contributions of individual features, describe the local decision pathway, and evaluate the instance-level behavior of the classifier. The numbers are used to represent major analysis steps that require sequential reasoning, whereas dashes are utilized to list supporting details under each major step. This guidance ensures that the interpretation remains methodical, informative, and aligned with XAI practices. Lastly, the contextual reference embeds relevant global feature importance information into the prompt. By providing this prior knowledge, the model can compare global patterns with local decision logic, thereby enhancing the depth and reliability of the interpretation.

## 4. Experimental Design

### 4.1. Dataset

To validate the effectiveness of the proposed method, we utilize the publicly available Car Hacking Dataset [33]. This dataset was constructed by capturing CAN traffic from a real vehicle via the on-board diagnostics II (OBD-II) port and includes multiple types of attack scenarios. Each entry in the dataset corresponds to a single CAN message and includes detailed information such as timestamps, CAN ID, data length code (DLC), and the 8-byte data field. Timestamp data supports temporal analysis by revealing the patterns of attack activity over time. CAN ID and DLC offer information about the targeted messages and their structure. The 8-byte data field, which serves as the input variables in this study, captures the payload value of each CAN message. Each byte within this field corresponds to values associated with specific vehicle components, including sensor readings, control commands, or status indicators. All input variables are expressed as integer values ranging from 0 to 255 and are directly utilized as input features for model training. As these values inherently represent physically meaningful properties of CAN messages, additional normalization is considered unnecessary. Furthermore, since the 8-byte data field comprehensively captures essential control information, feature selection is not performed. Table 3 summarizes the input variables used in the intrusion detection model.

The proposed method classifies CAN messages into five categories—Normal, DoS, Fuzzy, Gear, and revolutions per minute (RPM), as detailed in Table 4. The Normal category indicates standard CAN operations without malicious interference. The DoS attacks involve injection of messages with the CAN ID ‘0000’ at 0.3-millisecond intervals, overwhelming the CAN bus. Fuzzy attacks introduce random CAN IDs and payloads at 0.5-millisecond intervals, causing erratic system behavior. Gear attacks target gear-related CAN IDs by injecting messages at 1-millisecond intervals, leading to incorrect gear display on the dashboard. Similarly, RPM attacks manipulate engine RPM-related CAN IDs, also injected at 1-millisecond intervals, resulting in inaccurate RPM readings. In this study, we used a sampled subset of the dataset to reduce the computational cost while preserving the overall data distribution. The dataset comprises 701,832 Normal samples, 29,501 DoS attack samples, 24,624 Fuzzy attack samples, 29,944 Gear samples, and 32,539 RPM samples. It provides a diverse and balanced foundation for evaluating the performance of the proposed intrusion detection model. However, a class imbalance exists across the categories. While we acknowledge the presence of this imbalance, this study primarily focused on enhancing model interpretability and transparency rather than maximizing classification performance. Therefore, techniques to mitigate class imbalance, such as oversampling [43] and class balancing [44], were not applied in this study. For future work, we plan to explore methods such as the synthetic minority oversampling technique (SMOTE) [45] and class-weighted loss functions [44] to further improve classification performance and strengthen the reliability of interpretability under imbalanced conditions.

### 4.2. Experimental Settings

The performance of the proposed method was evaluated through comparative experiments with various ML-based prediction models [46]. The training, validation, and test datasets were split into 70%, 10%, and 20%, respectively. A total of 13 ML models were utilized for the experiments, including logistic regression [47], decision tree [48], random forest [49], multi-layer perceptron (MLP) Classifier [50], linear discriminant analysis [51], AdaBoost [52], ExtraTree [53], k-nearest neighbor [54], naive Bayes [55], gradient boosting [36], CatBoost [56], XGBoost [57], and LightGBM [24].

For the VLM utilized in Section 3.3 and Section 3.4, this study employed OpenAI GPT-4o [58]. GPT-4o is a multimodal large language model capable of processing and reasoning over both textual and visual inputs. It is designed to offer enhanced performance and lower latency compared to its predecessor, GPT-4 Turbo [59], while maintaining high accuracy across a range of multimodal tasks. Unlike unimodal models, GPT-4o has been specifically optimized to handle cross-modal alignment, enabling it to accurately interpret relationships between images and text.

In this study, the vision-language capabilities of GPT-4o were leveraged to analyze visual representations of model outputs, including feature importance plots, and generate detailed textual descriptions. This allows for a more interpretable explanation of ML model behavior, particularly when visual outputs should be converted into human-readable formats. In addition, the advanced natural language generation capabilities of GPT-4o contribute to producing coherent and contextually appropriate explanations, making it a suitable choice for supporting XAI in intrusion detection.

### 4.3. Evaluation Metrics

For the quantitative evaluation of the CAN ID model, we used precision, recall, F1 Score, and accuracy, which are commonly used in classification models [60]. Precision measures the proportion of correctly predicted positive instances, reflecting the model’s exactness. Recall quantifies the model’s ability to correctly identify positive instances among all actual positives. The F1 Score, the harmonic mean of precision and recall, provides a balanced evaluation when both metrics are important. Accuracy reflects the proportion of correctly predicted instances across the entire dataset, providing an overall measure of classification performance. Equations (1)–(4) present the mathematical definitions of these metrics.
$Precision=\frac{True\;Positive}{True\;Positive+False\;Positive}$.(1)$Recall=\frac{True\;Positive}{True\;Positive+False\;Negative}$.(2)$F1\;Score=2\times \frac{Precision\times Recall}{Precision+Recall}$.(3)$Accuracy=\frac{True\;Positive+True\;Negative}{True\;Positive+False\;Positive+False\;Negative+True\;Negative}$.(4)

For the qualitative evaluation of VLM-generated textual explanations, we conducted a manual review of the generated outputs based on their clarity, coherence, and alignment with the visualized feature importance results. We focused on assessing how effectively the VLM interprets and verbalizes key patterns observed in the graph. Specifically, we examined whether the descriptions clearly and accurately identified the most important features, provided logical reasoning consistent with the visual evidence, and maintained overall coherence and relevance to the model’s decision context. This structured qualitative analysis provides a systematic basis for evaluating the utility and reliability of VLM-generated textual explanations, supporting their potential application in XAI systems for in-vehicle CAN intrusion detection.

In addition to the manual analysis, a user study was conducted with ten researchers in the fields of communication and cybersecurity. Participants were asked to evaluate the VLM-generated textual explanations using a five-point Likert scale [61] across five criteria: usability, relevance, fluency, accuracy, and overall satisfaction. This expert-driven evaluation complements the manual review by incorporating domain-specific feedback, thereby offering further insight into the practical effectiveness and perceived quality of the proposed method.

## 5. Experimental Results

### 5.1. Intrusion Detection Performance

Based on the experimental setup described in Section 4.2, we evaluated the intrusion detection performance of 13 ML models for in-vehicle CAN intrusion detection. Table 5 presents a comparison of precision, recall, F1-score, and accuracy for all models. The best performing model for each metric is indicated in bold, while the second best is underlined.

Among them, the LightGBM-based intrusion detection model demonstrated the highest performance across all evaluation metrics. XGBoost and CatBoost, which are also gradient boosting-based ensemble models, followed closely, showing highly competitive results with marginal differences. These results underscore the effectiveness of boosting-based ensemble methods in modeling the complex patterns inherent in CAN intrusion data.

In contrast, traditional statistical models such as logistic regression and linear discriminant analysis yielded relatively low scores, particularly in recall and F1-score. This suggests their limited capacity to capture nonlinear relationships and temporal patterns inherent in CAN message sequences. Furthermore, AdaBoost exhibited the lowest performance across all metrics, likely due to its sensitivity to noise and reliance on comparatively weak base learners. The performance gap between AdaBoost and gradient boosting variants underscores the importance of model architecture in intrusion detection tasks.

Overall, the results confirm that modern ensemble models, particularly LightGBM, offer robust and accurate performance for in-vehicle CAN intrusion detection. Their capacity to handle high-dimensional, time-dependent CAN data makes them well-suited for real-world vehicle cybersecurity applications.

### 5.2. Vision-Language Model-Based Global Feature Importance Analysis for In-Vehicle CAN Intrusion Detection Model

**Figure 4:**
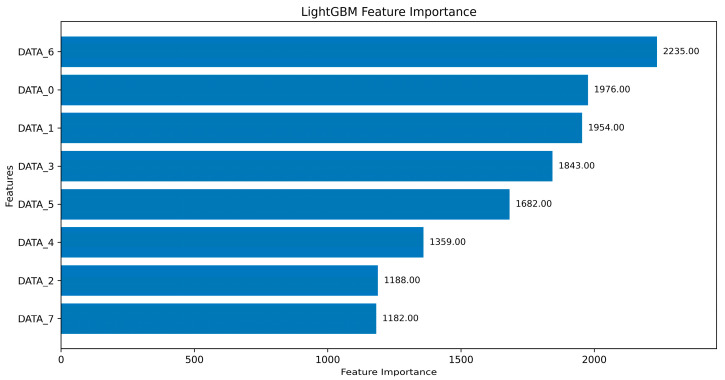
Global feature importance of proposed method.

We evaluated the capacity of the VLM to interpret global feature importance in textual form. Figure 4 presents the feature importance graph obtained from the trained LightGBM model. Feature importance was measured using the split criterion, which counts the number of times that a feature is used for data partitioning across all decision trees in the LightGBM model. Table 6 presents the corresponding textual explanation generated by the VLM using the structured prompt described in Table 1.

The interpretation accurately identified DATA_6, DATA_0, and DATA_1 as the three most influential features, highlighting their high contribution scores. The model emphasized that these byte-level input variables likely encode signal patterns or payload segments critical for distinguishing between normal and anomalous CAN traffic. Conversely, features including DATA_2 and DATA_7, which exhibited lower importance scores, were interpreted as carrying less discriminative information. This interpretation was supported by possible reasons such as low variability, redundancy, or reduced relevance in the model’s decision path. Importantly, the explanation adheres to the global nature of the analysis by avoiding association between individual features and specific intrusion types, including DoS and Gear. Instead, it focuses on the overall contribution of each input variable to the model’s multi-class classification performance, providing a general understanding of how the LightGBM model prioritizes certain byte positions in the CAN payload. The VLM further offers logical reasoning regarding the model’s prioritization strategy, including information-theoretic considerations and the statistical salience of high-ranking features.

These findings demonstrate the utility of VLMs in enhancing explainability by translating visual model outputs into human-readable narratives. The ability to automatically generate structured, domain-relevant explanations enables the users to better understand which features the model relies on and why. As a result, the proposed VLM-based method contributes to the development of interpretable and trustworthy intrusion detection systems for vehicle cyber-physical environments.

### 5.3. Vision-Lanaguage Model-Based LIME Analysis for In-Vehicle CAN Intrusion Detection Model

**Figure 5:**
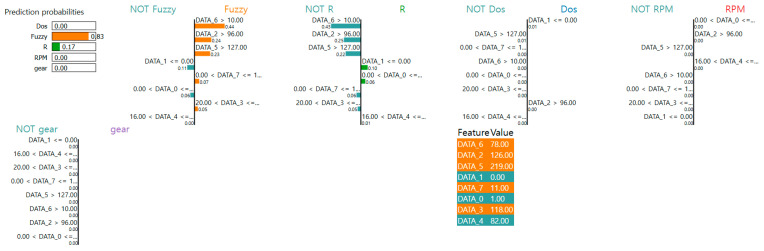
LIME plot of proposed scheme.

We conducted a detailed analysis of a VLM-generated textual interpretation based on the LIME visualization for a specific instance classified as “Fuzzy” with a prediction probability of 0.83. Figure 5 presents the LIME plot derived from the trained LightGBM model, and Table 7 displays the corresponding textual explanation generated by the VLM using the structured prompt described in Table 2.

The interpretation accurately identified DATA_6, DATA_2, and DATA_5 as the primary contributing features to the model’s decision. Among these, DATA_6 was correctly highlighted as the most important feature both globally and locally. This demonstrates the VLM’s capability to detect and articulate alignment between global and local perspectives. Furthermore, the explanation emphasized that DATA_2 and DATA_5 were not among the top global features but exerted significant local influence. This demonstrates the model’s ability to capture instance-specific variations in feature importance. The interpretation also addressed negatively contributing features, including DATA_1 and DATA_0, with the latter exhibiting low local importance despite its high global importance. This contrast was clearly articulated, supporting a nuanced understanding of how global trends may not always translate into local relevance. This can be shown as a significant motivation for applying LIME analysis in the interpretability framework.

In addition, the interpretation provided a coherent explanation of byte-level decision logic, referencing how specific value ranges across features activated class-specific thresholds. By capturing the rule-based nature of the LightGBM model, the VLM demonstrates an understanding of its underlying logic, thereby enhancing interpretability. Most notably, the interpretation effectively integrated the global feature importance into the instance-level reasoning. It evaluated the alignment and divergence between global and local patterns, addressing a key objective of the proposed method. The generated explanation was logically structured, technically sound, and expressed in a formal tone suitable for reporting.

These findings confirm that the VLM, when guided by a structured prompt, can generate explanations that are not only consistent with the visual LIME output but also analytically rich and aligned with the goal of XAI. This suggests strong potential for using the VLM in automated interpretability pipelines, particularly in safety-critical domains such as vehicle cybersecurity, where reliable and explainable model behavior is essential. The performance of the proposed method across different attack types can be found in Supplementary Materials.

### 5.4. Ablation Study

To verify the effectiveness of the proposed method, we conducted an ablation study. We examined how the VLM interprets LIME analysis results in the absence of global feature importance information. Table 8 presents the textual interpretation generated by the VLM using only local LIME output without access to global feature importance as contextual reference.

Compared to the interpretation generated by our proposed method in Table 7, the explanation in Table 8 exhibits several limitations. While both analyses identify DATA_6, DATA_2, and DATA_5 as dominant contributors to the Fuzzy classification, the LIME-only interpretation fails to contextualize the relative importance of these features in the model’s global behavior. For instance, the absence of global reference leads to an underestimation of the discrepancy between DATA_0’s high global importance and its relatively low contribution in this instance. As a result, the explanation lacks the ability to distinguish whether locally influential features are also globally significant or merely relevant due to instance-specific patterns.

Moreover, the LIME-only explanation does not explicitly reflect on the consistency and divergence between local and global model behavior. This restricts the analytical depth and limits the user’s ability to validate the model’s reasoning process from a broader perspective. In contrast, our proposed method integrates global feature importance as prior knowledge, enabling the VLM to provide a more comprehensive and balanced interpretation that captures both dataset-level patterns and instance-specific reasoning.

These results demonstrate that incorporating global context significantly improves the clarity, reliability, and interpretive depth of LIME-based explanations. The comparison validates the effectiveness of our method, which supports more trustworthy and explainable intrusion detection for in-vehicle CAN. Additional results of the ablation study can be found in Supplementary Materials.

### 5.5. Qualitative Evaluation

A five-point Likert scale survey was conducted to systematically evaluate user satisfaction with the proposed method. The survey was completed by ten participants. The scale consisted of the following response options: very dissatisfied, dissatisfied, neutral, satisfied, and very satisfied. This format provides a balanced range of positive and negative choices, allowing respondents to give clear and meaningful feedback. As summarized in Table 9, the evaluation was performed across five criteria: usability, relevance, fluency, accuracy, and overall satisfaction.

Overall, the survey results indicate a favorable response to the proposed method. Across all five criteria, the majority of responses fell within the satisfied and very satisfied categories, demonstrating a generally high level of user satisfaction. Among these criteria, usability and overall satisfaction received the most positive ratings, with over 80% of respondents indicating satisfaction. Responses for relevance, fluency, and accuracy were slightly more varied, with some participants selecting neutral or dissatisfied options. Nevertheless, the majority of responses in these categories still fell within the satisfied or very satisfied range, indicating an overall positive perception. These findings suggest that the VLM-generated textual explanations were perceived as useful, accurate, and well-aligned with the visual outputs, supporting the effectiveness of the proposed method in practical applications.

## 6. Conclusions

In this study, we proposed a novel method to enhance the explainability of an in-vehicle CAN intrusion detection model by integrating a VLM with LIME analysis. Among various predictive models evaluated, the ML model that exhibited the best performance on the given vehicle CAN dataset was selected for further analysis. To capture both global and local interpretability, we first utilized the VLM to generate textual descriptions of global feature importance. Subsequently, these global features were incorporated as prior knowledge to guide the VLM in producing instance-level explanations based on LIME analysis, offering detailed insights into the model’s decision-making process.

Experimental results confirmed that LightGBM outperformed other ML models in classification accuracy. The VLM-based LIME analysis revealed that the global features with the highest importance were DATA_6, DATA_0, and DATA_1. In contrast, for a particular data instance classified as the Fuzzy class, the most influential local features were identified as DATA_6, DATA_2, and DATA_5. By converting the LIME visualization into a structured textual explanation, the proposed method allows for a more transparent interpretation that considers the relationship between global and local feature importance.

These findings demonstrate the potential of the VLM-assisted explanation method to support advancements in XAI. In particular, as AI-driven intrusion detection techniques for in-vehicle CAN have garnered increased attention, vehicle manufacturers are expected to actively adopt big data-driven cybersecurity solutions. This research contributes to the digital transformation of the vehicle cybersecurity industry by enhancing model transparency and trustworthiness.

Although the VLM-assisted method demonstrated strong interpretability performance, VLM processing may impose significant computational burdens, particularly in resource-constrained in-vehicle environments. Therefore, future work will focus on optimizing the VLM architecture to enhance computational efficiency. This includes exploring model compression techniques and lightweight adaptation strategies to facilitate the real-time deployment of VLM-based explanation systems without compromising interpretability [62]. In particular, we plan to benchmark lightweight, self-hosted VLMs such as MiniGPT-4 [63] and MobileVLM [64] to enable more efficient, low-latency inference suitable for practical in-vehicle applications.

Moreover, while LIME analysis was adopted as the primary XAI technique in this study, other post hoc methods such as SHAP, PDP, and counterfactual explanations also offer valuable interpretability perspectives. Future work will explore integrating various XAI techniques with VLM to improve the overall interpretability of ML models. Additionally, considering the prompt sensitivity of VLM outputs, subsequent studies will focus on designing adaptive prompt templates tailored to each input instance and corresponding visual output, thereby improving the accuracy, relevance, and consistency of generated explanations.

## Figures and Tables

**Table 1:** Template for prompt in analyzing the graph visualization result of global feature importance in machine learning model.

Type	Template
Role description	You are a domain expert in machine learning and XAI. Your objective is to analyze the global feature importance of a trained machine learning model and produce precise and insightful textual explanations suitable for the report.
Task specification	<Task> - The image shows the global feature importance graph of {Prediction model} model used for intrusion detection in a vehicle CAN. - This is a multi-class classification task that classifies CAN messages into five categories: Normal, DoS, Fuzzy, Gear, and RPM.- Input features, DATA_0 to DATA_7, represent byte-level values from CAN messages. - In the graph, the *Y*-axis represents the input variables (DATA_0 to DATA_7), and the *X*-axis represents their corresponding importance scores. - The numerical values displayed at the end of each bar indicate the feature importance score calculated by the model, reflecting each feature’s contribution to the overall prediction performance. </Task>
Interpretation guideline	<Interpretation guideline> - Please provide a structured explanation that includes: 1. A summary of the top three most important features, including their relative importance scores. 2. A discussion on the contribution of these features to detecting specific intrusion types. 3. An interpretation of lower-ranked features and potential reasons for their limited impact. 4. A logical analysis of how the model prioritizes input variables in the context of CAN message classification. - Since the provided graph represents global feature importance across all classes, do not associate specific input features with particular intrusion types. - Focus on the overall contribution of each feature to the model’s general classification performance. - The explanation should be clear, analytical, and suitable for inclusion in a research paper. - Use technical language appropriate for a machine learning and cybersecurity audience. </Interpretation guideline>

**Table 2:** Template for prompt in analyzing the graph visualization result of LIME analysis.

Type	Template
Role description	You are a domain expert in machine learning and explainable artificial intelligence. Your objective is to analyze the local interpretable LIME visualization for a specific data instance classified by a vehicle CAN intrusion detection model, and to produce a precise and insightful textual explanation suitable for inclusion in a report.
Task Specification	<Task> - The image shows the LIME explanation for a specific instance classified by {Prediction model} model trained to detect intrusions in a vehicle CAN. - This is a multi-class classification task that classifies CAN messages into five categories: Normal, DoS, Fuzzy, Gear, and RPM. - Input features, DATA_0 to DATA_7, represent byte-level values from CAN messages. -The LIME visualization consists of: - A bar plot of prediction probabilities, indicating the model’s confidence across the five target classes. - In this specific case, the instance was classified as class {Predicted class} with the highest probability of {Highest probability value}. - A decision path showing feature-based rules that influenced the prediction. - Each horizontal bar represents a feature condition that was satisfied for this input instance. - The value on the right side of each bar indicates the local contribution (weight) of that condition to the final class prediction. - Positive contribution values push the prediction toward the predicted class, while negative values push it away. - Longer bars indicate greater influence on the prediction decision. - A list of the actual input feature values for this instance. - The following LIME feature conditions and contribution values are observed for this instance: {LIME feature conditions and contribution values} </Task>
Interpretation guideline	<Interpretation guideline> - Please provide a structured explanation that includes: 1. A summary of the most influential features in the local explanation, including both positively and negatively contributing features, and their corresponding contribution values. 2. A discussion on how the model locally prioritizes features to predict the target class for this instance. 3. An interpretation of features with minimal or near-zero contribution and possible reasons for their limited relevance. 4. A logical analysis of the local decision-making process as revealed by LIME in the context of byte-level classification of CAN messages. 5. A reflection on whether the local feature importance observed in the LIME explanation aligns with or diverges from the global feature importance patterns. An analysis of what this reveals about the model’s instance-specific decision behavior. - Focus on the local behavior of the model for this instance only. Do not generalize feature relevance across the entire dataset. - The explanation should be clear, analytical, and suitable for inclusion in a research paper. - Use technical language appropriate for a machine learning and cybersecurity audience. </Interpretation guideline>
Contextual reference	<Contextual reference> - Use the following result of global feature importance analysis to complement your interpretation: {Result of global feature importance analysis of vehicle CAN intrusion detection model} - In your explanation, you may reflect on whether the local importance shown in the LIME result aligns with or diverges from the global importance patterns, and what this reveals about the model’s behavior on this specific instance. </Contextual reference>

**Table 3:** Input variables of Car Hacking Dataset.

Input Variable	Variable Type	Description
DATA_0	Continuous	Byte-level payload values (0–255) of CAN message
DATA_1		
DATA_2		
DATA_3		
DATA_4		
DATA_5		
DATA_6		
DATA_7		

**Table 4:** Output variables of Car Hacking Dataset.

Output Variable	Variable Type	No. of Class	Description	Class	Samples
Vehicle intrusion	Categorical	5	Type of the vehicle intrusion	Normal	701,832
				DoS	29,501
				Fuzzy	24,624
				Gear	29,944
				RPM	32,539

**Table 5:** Intrusion detection performance of the in-vehicle CAN intrusion detection model.

Model	Precision	Recall	F1 Score	Accuracy
Logistic Regression	0.7893	0.7548	0.7692	0.9571
Decision Tree	0.9014	0.9913	0.9302	0.9652
Random Forest	0.9015	0.9919	0.9305	0.9652
MLP Classifier	0.9013	0.9914	0.9302	0.9652
Linear Discriminant Analysis	0.7106	0.7333	0.7117	0.9370
AdaBoost	0.3993	0.5404	0.3961	0.6702
ExtraTree	0.9015	0.9919	0.9306	0.9653
K-Nearest Neighbor	0.7919	0.7990	0.7953	0.9640
Naïve Bayes	0.8992	0.9421	0.9011	0.9575
Gradient Boosting	0.9015	0.9912	0.9302	0.9652
CatBoost	0.9015	0.9918	0.9305	0.9653
XGBoost	0.9015	0.9919	0.9305	0.9653
LightGBM	**0.9015**	**0.9919**	**0.9306**	**0.9653**

**Table 6:** Textual interpretation of global feature importance using vision-language model.

**Result**
**Global Feature Importance Analysis of LightGBM for CAN Intrusion Detection** **1. Summary of Top Three Most Important Features** The global feature importance plot of the LightGBM model, trained to classify CAN messages into five categories (Normal, DoS, Fuzzy, Gear, and RPM), reveals the following top three features based on their contribution to the overall model performance: • **DATA_6**: With an importance score of **2235**, this is the most influential feature. Its prominence suggests that byte-level values from this field carry significant discriminative patterns used by the model in classifying CAN traffic. • **DATA_0**: Ranked second with a score of **1976**, DATA_0 also contributes substantially to the decision-making process, indicating its relevance in capturing structural or behavioral distinctions across message types. • **DATA_1**: This feature has a score of **1954**, marginally lower than DATA_0, suggesting a comparable level of importance. The model likely leverages subtle interdependencies between these bytes for robust multi-class classification. Together, these three features account for a substantial portion of the total feature importance, indicating that specific byte positions in CAN payloads are consistently informative across different intrusion types. **2. Contribution of Top Features to Intrusion Detection** While this analysis considers **global feature importance** and does not attribute features to specific attack types, the high importance of DATA_6, DATA_0, and DATA_1 implies that these fields capture generalizable patterns that differentiate both benign and malicious behaviors. These bytes may encode information sensitive to protocol misuse or anomalous behaviors common across various attack categories such as DoS or fuzzing. Their dominance suggests that the model detects deviations or patterns in these byte positions that are statistically distinct from normal traffic across multiple classes. **3. Interpretation of Lower-Ranked Features** The remaining features—**DATA_3 (1843)**, **DATA_5 (1682)**, **DATA_4 (1359)**, **DATA_2 (1188)**, and **DATA_7 (1182)**—though still contributing to model performance, exhibit decreasing importance. This suggests several possible interpretations: • These features may encode information that is **less discriminative** across all classes, possibly because their values exhibit **higher variance within classes** or **less consistent patterns** across different types of traffic. • It is possible that some of these fields represent **redundant or correlated information**, where the model relies more heavily on the most informative bytes (e.g., DATA_0–DATA_3) and uses the others to refine predictions. • Features like DATA_2 and DATA_7, having the lowest scores (~1180), may be **less frequently activated** in decision paths or may only contribute to a limited subset of classification boundaries. **4. Model Prioritization Logic in CAN Message Classification** The LightGBM model’s feature prioritization reflects an **information-theoretic optimization** where byte positions that consistently help reduce entropy (i.e., uncertainty in class prediction) are ranked higher. In the context of CAN message classification, certain byte positions (e.g., DATA_6, DATA_0) likely align with payload segments that encapsulate **control-critical or attacker-manipulable** fields. These positions may correlate with identifiers or command values that are disproportionately affected during attacks, making them statistically salient. Conversely, lower-ranked features may correspond to **non-critical bytes** or fields that remain invariant across traffic conditions, thus offering limited utility for classification. The distribution of importance suggests that the model effectively distinguishes signal-carrying bytes from noise, optimizing the feature space to prioritize high-signal content in the multi-class setting. **Conclusion** The global feature importance analysis confirms that the LightGBM model effectively identifies and exploits specific byte-level fields (particularly DATA_6, DATA_0, and DATA_1) that contribute most significantly to intrusion detection across all CAN message classes. The declining importance of other features reflects the model’s ability to focus on the most predictive information, supporting efficient and interpretable decision-making in a complex multi-class classification task. This prioritization is critical for deploying lightweight and explainable intrusion detection systems in automotive cyber-physical environments.

**Table 7:** Textual interpretation of LIME analysis using vision-language model.

**Result**
**Local Explanation of LIME for CAN Intrusion Detection—Class “Fuzzy”** **1. Summary of the Most Influential Features** The LIME explanation reveals that the instance was classified as **“Fuzzy”** with the highest predicted probability of **0.83**. The following features had the most significant contributions: • **Positive contributors:** ○ DATA_6 > 10.00 (**+0.4373**): This was the most influential positive contributor, strongly supporting the “Fuzzy” classification. ○ DATA_2 > 96.00 (**+0.2443**): Reinforces the prediction, indicating this feature value aligns with patterns seen in Fuzzy-labeled messages. ○ DATA_5 > 127.00 (**+0.2265**): Also significantly pushed the prediction toward “Fuzzy”. ○ 0.00 < DATA_7 ≤ 109.00 (**+0.0684**) and 20.00 < DATA_3 ≤ 137.00 (**+0.0488**) added modest support to the prediction. • **Negative contributors:** ○ DATA_1 ≤ 0.00 (**−0.1055**): Opposed the prediction, potentially aligning with patterns common in other classes. ○ 0.00 < DATA_0 ≤ 8.00 (**−0.0608**) slightly reduced the confidence in classifying the instance as “Fuzzy”. ○ 16.00 < DATA_4 ≤ 107.00 (**−0.0047**) had negligible negative impact. Overall, the decision was largely driven by strong signals from DATA_6, DATA_2, and DATA_5. **2. Local Feature Prioritization Logic** The model prioritized features that exhibited high predictive relevance under the given input conditions. Specifically: • DATA_6, the highest globally ranked feature, was also the strongest local contributor. Its value (78.0) satisfied the threshold (>10.00), making it highly influential. • DATA_2 and DATA_5, although lower-ranked globally, had substantial local contributions due to satisfying their class-specific conditions. • Features such as DATA_7 and DATA_3 contributed positively, albeit with lower impact. This shows that the model leverages a localized rule chain, activating only a subset of features whose values align with pre-learned patterns for “Fuzzy”. **3. Interpretation of Low-Contribution Features** • DATA_4 had a minimal contribution (**−0.0047**), suggesting its value was neutral or uninformative in this context. • DATA_0, though ranked second in global importance, had only a minor negative impact in this instance (−0.0608). This suggests its value did not strongly align with “Fuzzy”-specific patterns here. • DATA_1 showed moderate negative contribution (−0.1055), reflecting a possible conflict between the instance and typical “Fuzzy” profiles. These observations indicate that high global importance does not guarantee local relevance—a key insight enabled by LIME. **4. Byte-Level Local Decision Logic** The model’s decision reflects how specific byte-level thresholds are used to distinguish among attack classes. In this case: • The combination of moderate-to-high values in DATA_6, DATA_2, and DATA_5 forms a strong local signal for class “Fuzzy”. • Negatively contributing features like DATA_1 and DATA_0 did not satisfy Fuzzy-specific rules, slightly reducing the classification confidence. LIME’s decision path illustrates a model behavior that is rule-based, interpretable, and tightly linked to threshold logic, typical in gradient-boosted tree models like LightGBM. **5. Alignment with Global Feature Importance** The local importance mostly aligns with global importance rankings: • DATA_6 is globally most important and also the dominant local feature. • However, DATA_0, despite its global prominence, played a minor and even negative role here, showing that its utility is instance-dependent. • Conversely, DATA_5 and DATA_2, which are moderate to low in global ranking, emerged as significant locally—highlighting their conditional relevance to specific instances. This underscores the complementary nature of global and local interpretability: global importance reveals general patterns, while LIME exposes how these patterns apply (or don’t) in specific decisions. **Conclusion** The LIME-based analysis of this CAN message classified as “Fuzzy” demonstrates how a small subset of byte-level features, particularly DATA_6, DATA_2, and DATA_5, drove the local prediction. While the result is partially consistent with global importance rankings, discrepancies like the limited local impact of DATA_0 emphasize the need for instance-level interpretability. This kind of hybrid analysis is essential for developing trustworthy, explainable, and safety-critical intrusion detection models in vehicular environments.

**Table 8:** Textual interpretation of LIME analysis except global feature importance using vision-language model.

**Result**
**Local Explanation of LIME for CAN Intrusion Detection—Class “Fuzzy”** **1. Summary of the Most Influential Features** In this case, the instance was classified as **Fuzzy** with the **highest prediction probability of 0.83**, indicating high model confidence. The LIME explanation reveals the local decision process by attributing contributions of feature conditions to the predicted class (**Fuzzy**). The most influential features, ranked by absolute contribution magnitude, are as follows: • **Positive contributors:** ○ DATA_6 > 10.00: **+0.4373**—Strongest positive contributor; suggests this byte-level value is highly indicative of Fuzzy-class behavior. ○ DATA_2 > 96.00: **+0.2443**—Also significant; supports anomalous patterns associated with the Fuzzy class. ○ DATA_5 > 127.00: **+0.2265**—Further reinforces the prediction, aligning with statistical patterns learned from Fuzzy-class data. ○ 0.00 < DATA_7 <= 109.00: **+0.0684** ○ 20.00 < DATA_3 <= 137.00: **+0.0488** • **Negative Contributions** ○ DATA_1 <= 0.00: **−0.1055**—Moderately suppresses the Fuzzy prediction; potentially a signal more common in other classes ○ 0.00 < DATA_0 <= 8.00: **−0.0608** ○ 16.00 < DATA_4 <= 107.00: **−0.0047**—Very minimal impact, nearly neutral. **2. Local Feature Prioritization Logic** The local model prioritization clearly emphasizes high values of DATA_6, DATA_2, and DATA_5 as indicative of Fuzzy intrusions. These byte-level features may capture payload anomalies or entropy patterns associated with injected random/fuzzy CAN frames. Notably, the model’s reliance on DATA_6 > 10.00 as the dominant rule (contribution: +0.4373) suggests that this byte is particularly sensitive to Fuzzy behavior in the training data distribution. The use of threshold-based binning (e.g., DATA_2 > 96.00, DATA_5 > 127.00) reflects the model’s learned boundaries that separate Fuzzy instances from other types. This is consistent with decision tree behavior within LightGBM, where split thresholds are optimized during training to maximize information gain. **3. Interpretation of Low-Contribution Features** • 16.00 < DATA_4 <= 107.00: **−0.0047**—While this feature condition was satisfied, it exerted negligible influence on the decision. This may indicate that DATA_4 contributes more globally across classes rather than being distinctive for Fuzzy instances. • Similarly, although DATA_0 and DATA_7 were involved, their impact was modest, possibly due to their distribution overlap across multiple classes. These minimal contributions could also be due to redundancy with more dominant features or lower mutual information with the target class in the local region of the feature space. **4. Byte-Level Local Decision Logic** This instance’s classification as **Fuzzy** results from the model identifying a distinctive combination of high byte values in critical features (DATA_6, DATA_2, DATA_5) that align with fuzzing attack signatures. These patterns may represent payload anomalies generated by random data injection common in Fuzzy attacks. The model effectively discounts certain conditions (e.g., DATA_1 <= 0.00, DATA_0 low values) that may be more associated with normal or Gear messages. The presence of both positive and negative contributions illustrates the model’s nuanced decision boundary, balancing evidence for and against the Fuzzy class within the local context. **Conclusion** Overall, this LIME explanation offers interpretability into the LightGBM classifier’s internal logic, highlighting how specific byte-level thresholds guide localized decisions. This is especially valuable in cybersecurity settings where transparency and trust in intrusion detection systems are critical.

**Table 9:** Evaluation of proposed method through the survey.

Metric	Very Dissatisfied	Dissatisfied	Neutral	Satisfied	Very Satisfied
Usability	0.0%	0.0%	20.0%	60.0%	20.0%
Relevance	0.0%	10.0%	20.0%	60.0%	10.0%
Fluency	0.0%	0.0%	30.0%	60.0%	10.0%
Accuracy	0.0%	10.0%	30.0%	50.0%	10.0%
Overall satisfaction	0.0%	0.0%	20.0%	60.0%	20.0%

## Data Availability

https://ocslab.hksecurity.net/Datasets/car-hacking-dataset (accessed on 1 March 2025).

## Supplementary Materials

The following supporting information can be downloaded at https://www.mdpi.com/article/10.3390/s25103020/s1, Figure S1: LIME plot of proposed scheme—classified as RPM class; Figure S2. LIME plot of proposed scheme—classified as Normal class; Figure S3. LIME plot of proposed scheme—classified as DoS class; Table S1. Textual interpretation of LIME analysis using vision-language model—corresponding to Figure S1; Table S2. Textual interpretation of LIME analysis using vision-language model—corresponding to Figure S2; Table S3. Textual interpretation of LIME analysis using vision-language model—corresponding to Figure S3; Table S4. Textual interpretation of LIME analysis except global feature importance using vision-language model—corresponding to Figure S1; Table S5. Textual interpretation of LIME analysis except global feature importance using vision-language model—corresponding to Figure S2; Table S6. Textual interpretation of LIME analysis except global feature importance using vision-language model—corresponding to Figure S3.
