# Supplementary material for: Vision-Language Model-Based Local Interpretable Model-Agnostic Explanations Analysis for Explainable In-Vehicle Controller Area Network Intrusion Detection
Source: Sensors (Basel). 2025 May 10;25(10):3020. doi: 10.3390/s25103020 (PMC12115109; doi:10.3390/s25103020)
Supplement: Supplementary file 1 [file sensors-25-03020-s001.zip › sensors-3602370-supplementary.pdf]

*Article*

# Supplementary Materials to: Vision-Language Model-Based Local Interpretable Model-Agnostic Explanations Analysis for Explainable In-Vehicle Controller Area Network Intrusion Detection


**Jaeseung Lee [1] and Jehyeok Rew [2,*]**

[1] School of Electrical Engineering, Korea University, Seoul 02841, Republic of Korea; jason2133@korea.ac.kr
[2] Department of Data Science, Duksung Women's University, Seoul 01370, Republic of Korea
* Correspondence: jhrew@duksung.ac.kr


## Overview

This supplementary material provides additional results of proposed method in Section A. We also provide additional results of ablation study in Section B.

## A. Additional Results of Proposed Method

This section presents how the proposed method interprets LIME analysis results for three specific data instances, covering RPM, normal, and DoS. For each instance, the corresponding feature importance visualizations and VLM-generated textual explanations are provided. The experimental findings demonstrate that the proposed method consistently achieves robust interpretability performance across different attack types. Notably, even when interpreting a noisy instance that was classified as DoS with only a marginal decision boundary, the method maintains high robustness, highlighting its reliability under challenging conditions. These results confirm the strong generalization capability of the proposed method.

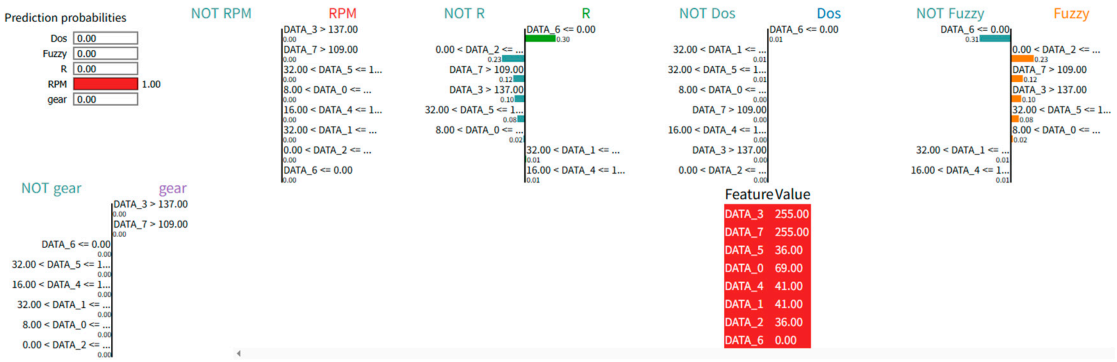


**Figure S1.** LIME plot of proposed scheme – classified as RPM class

**Table S1.** Textual interpretation of LIME analysis using vision-language model – corresponding to Figure S1

**Result**

**Local Explanation Analysis for CAN Intrusion Detection (Instance Classified as RPM)**

**1. Summary of Most Influential Features**

The LIME explanation reveals the following **eight features** contributed to the model's classification of this instance as **RPM**, with all contributions being positive, thus reinforcing the prediction:

| Feature Condition | Contribution Value |
|---|---|
| DATA_3 > 137.00 | +0.001502 |
| DATA_7 > 109.00 | +0.001485 |
| 32.00 < DATA_5 ≤ 127.00 | +0.001445 |
| 8.00 < DATA_0 ≤ 69.00 | +0.001358 |
| 16.00 < DATA_4 ≤ 107.00 | +0.001279 |
| 32.00 < DATA_1 ≤ 69.00 | +0.001266 |
| 0.00 < DATA_2 ≤ 96.00 | +0.001170 |
| DATA_6 ≤ 0.00 | +0.000642 |

All feature conditions positively influenced the final decision toward **RPM** classification, with no observed negative (contradictory) contributions.

**2. Model's Local Feature Prioritization Logic**

The model locally prioritized feature conditions that indicate specific value ranges or thresholds for particular bytes:

- **DATA_3 and DATA_7**: The largest contributions were associated with these two features exceeding relatively high thresholds (137.00 and 109.00 respectively), indicating that the payload's high byte values in these positions are strong signatures for RPM-related behavior.
- **DATA_5, DATA_0, DATA_4, DATA_1**: Intermediate-value ranges (between 8.00 and 127.00) for these bytes played meaningful roles. These conditions suggest that the instance conforms to expected byte patterns associated with legitimate RPM-related messages.
- **DATA_2**: Its moderate positive contribution (0.001170) shows it supported the classification, although it is globally ranked lower in importance.
- **DATA_6**: The instance's value for DATA_6 was 0.00, and although globally this is the most important feature overall, locally its contribution was relatively small (0.000642). Nevertheless, it still reinforced the decision.

Overall, the model combined multiple confirming byte conditions to robustly classify the message into the RPM class.

**3. Interpretation of Features with Minimal Contribution**

Although **DATA_6** is globally the most important feature, it had **the lowest local contribution** (0.000642) among the influential features for this instance. Possible reasons:

- **Low Activation**: The condition "DATA_6 ≤ 0.00" may be a common characteristic across multiple classes, thereby offering less discriminative power for this specific instance.
- **Contextual Redundancy**: Other features (DATA_3, DATA_7, DATA_5) provided stronger, more distinctive evidence for RPM classification, reducing reliance on DATA_6 locally.

No features exhibited negative contributions, meaning that the instance was internally consistent with the decision boundary for RPM.

**4. Logical Analysis of Local Decision-Making**

The local decision process reflects a **cumulative evidence-based strategy**:

- The LightGBM model did not depend on a single dominant feature but rather aggregated small, positive contributions across multiple feature conditions.
- The conditions involve **high or intermediate byte values**, suggesting that RPM messages are characterized by specific high-load or mid-range payload signatures.
- The narrow contribution margins (all around ~0.001–0.0015) indicate that this instance fits the RPM profile with high certainty but not due to extreme singular indicators—rather, a robust multi-feature match.

This fits LightGBM's behavior: combining multiple weak but coherent decision splits to produce confident classifications.

**5. Reflection on Local vs Global Importance Patterns**

Comparison with the global feature importance analysis:

| **Aspect** | **Observation** |
|---|---|
| DATA_6 | Globally ranked #1 but locally minor contributor. |
| DATA_0, DATA_1 | High global importance; moderately high local contributions. |
| DATA_3, DATA_5, DATA_4 | Lower global importance but **stronger local activation** for this instance. |
| DATA_2, DATA_7 | Lower global importance, still locally supportive. |

**Key Insight**:

While globally important features (DATA_0, DATA_1) remain relevant locally, **some globally lower-ranked features (DATA_3, DATA_5)** became crucial in this specific decision.

This divergence suggests that **the model dynamically adapts its reliance on features** based on instance-specific patterns. It does not rigidly prioritize features globally but tailors decision paths to each input's unique characteristics — a hallmark of effective gradient-boosted tree behavior.

**Conclusion**

The LIME local explanation highlights that the LightGBM model classified this instance as RPM with perfect confidence (1.00 probability) by **aggregating moderate positive evidence across several byte-level features**, rather than relying on a single dominant indicator.

The **dynamic reprioritization of features** — using globally lower-ranked features like DATA_3 and DATA_5 more heavily for this instance — underscores the model's flexibility and fine-grained decision-making capability.

This instance-specific analysis complements the global importance trends, providing deeper assurance about the model's robustness and adaptability in the multi-class CAN intrusion detection task.

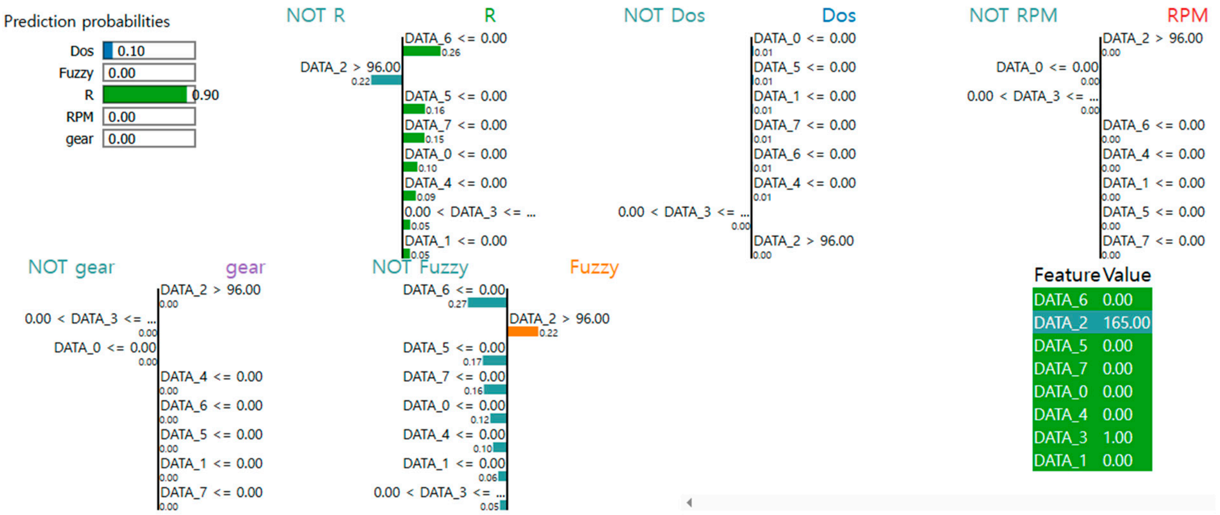


**Figure S2.** LIME plot of proposed scheme – classified as Normal class

**Table S2.** Textual interpretation of LIME analysis using vision-language model – corresponding to Figure S2

**Result**

**Local Explanation Analysis for CAN Intrusion Detection (Instance Classified as Normal)**

**1. Summary of Most Influential Features**

The LIME explanation highlights the following feature contributions for this specific instance:

**Positively contributing features** (supporting the classification as "Normal"):

- **DATA_6 ≤ 0.00** → Contribution: **+0.2725**
- **DATA_5 ≤ 0.00** → Contribution: **+0.1693**
- **DATA_7 ≤ 0.00** → Contribution: **+0.1617**
- **DATA_0 ≤ 0.00** → Contribution: **+0.1167**
- **DATA_4 ≤ 0.00** → Contribution: **+0.0979**
- **DATA_1 ≤ 0.00** → Contribution: **+0.0621**
- **0.00 < DATA_3 ≤ 20.00** → Contribution: **+0.0505**

**Negatively contributing feature** (opposing the classification as "Normal"):

- **DATA_2 > 96.00** → Contribution: **-0.2153**

Thus, the model's decision is primarily supported by DATA_6, DATA_5, DATA_7, and DATA_0, while DATA_2 introduces a counteracting influence.

**2. Model's Local Feature Prioritization**

At the local decision level:

- **DATA_6** emerges as the most influential feature. The condition **DATA_6 ≤ 0.00** strongly promotes the classification into the "Normal" class, consistent with its global importance ranking (#1 globally).
- **DATA_5**, **DATA_7**, and **DATA_0** also significantly reinforce the prediction toward "Normal", indicating that the absence or low byte values in these positions (0.00 or close to 0.00) are considered normal behavioral patterns.
- The **negative contribution of DATA_2** suggests that an unusually high value at this byte (165.00) contradicts the model's expectation for normal traffic. Nevertheless, this negative evidence is insufficient to overturn the dominant positive evidence.

This localized prioritization shows that the model heavily weighs zero or near-zero conditions in critical features to determine normalcy.

**3. Interpretation of Features with Minimal or Near-Zero Contribution**

Features such as **DATA_3** (although contributing positively) exhibit relatively low influence compared to DATA_6 or DATA_5. Its contribution (+0.0505) is smaller, suggesting:

- It plays a **secondary, supportive** role in confirming the prediction.
- DATA_3 conditions (being within a low range) align with normal behavior but are not individually decisive.

Other features not explicitly highlighted in the LIME decision path (e.g., possible higher-order interactions involving lower-ranked features) likely had negligible contribution because:

- Their byte values may not deviate sufficiently from norms.
- They lack statistically significant patterns in this instance.

**4. Logical Analysis of Local Decision-Making Process**

The local decision logic, as revealed by LIME, follows a **pattern-based discrimination** in which:

- **Zero or minimal byte values across key positions (DATA_6, DATA_5, DATA_7, DATA_0)** are interpreted as strong indicators of a normal CAN message.
- **Exceptionally high values**, as in DATA_2 (>96.00), are typically red flags but require stronger corroborative evidence across other features to alter the classification.

Thus, the model applies a **majority evidence principle**: the cumulative positive support from multiple features outweighs isolated signs of abnormality.

In byte-level CAN classification, this behavior is highly rational. It mirrors how cybersecurity analysts consider anomalies significant only when corroborated by multiple inconsistent signals rather than isolated outliers.

**5. Reflection on Alignment with Global Feature Importance**

The local behavior **largely aligns** with global feature importance patterns:

- **DATA_6**: Highest global importance → also the strongest local positive contributor (+0.2725).
- **DATA_0** and **DATA_1**: Ranked 2nd and 3rd globally → contribute positively at the local level as well.
- **DATA_2**, globally among the least important features, acts **negatively** in this instance, indicating that it can be locally significant when its value is extreme (here, 165.00).

This instance-specific decision behavior highlights that:

- **Global importance reflects potential**, not absolute local relevance.
- Even lower-ranked features (like DATA_2) can exert substantial localized influence when they present **anomalous conditions**.
- The model displays **adaptive reasoning**, dynamically adjusting feature weightings based on instance-specific patterns rather than rigidly following global rankings.

Such flexibility is crucial for achieving **robust, context-sensitive intrusion detection** in automotive networks where localized anomalies can vary widely.

**Conclusion**

The LIME explanation for this instance demonstrates that the LightGBM CAN intrusion detection model combines strong positive evidence from highly important global features (DATA_6, DATA_5, DATA_7, DATA_0) while carefully weighing negative evidence from DATA_2. The local decision prioritization aligns with the global feature ranking, reinforcing confidence in the model's consistency. Furthermore, the model's ability to adaptively integrate minor yet critical negative cues (DATA_2 high value) without overreacting showcases its maturity in handling real-world noisy data typical of CAN environments.

This nuanced local behavior is essential for ensuring explainable, reliable, and resilient

intrusion detection in safety-critical automotive applications.

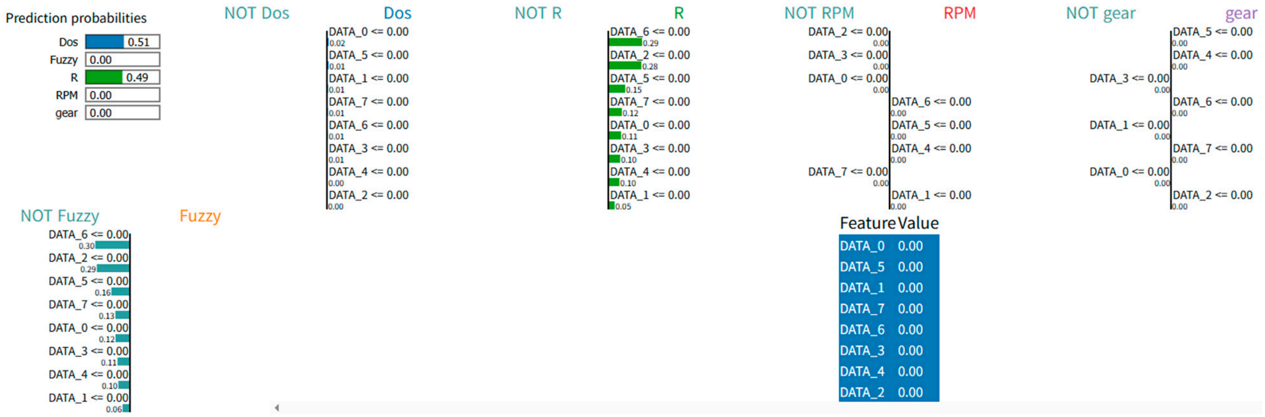


**Figure S3.** LIME plot of proposed scheme – classified as DoS class

**Table S3.** Textual interpretation of LIME analysis using vision-language model – corresponding to Figure S3

**Result**

**LIME-Based Local Interpretation for a DoS-Classified CAN Message Instance**

**1. Summary of the Most Influential Features**

The LIME explanation for the selected CAN message instance, classified as **DoS** with a prediction probability of **0.51**, highlights the following most influential features and their corresponding positive contributions to the classification:

| Feature | Condition Satisfied | Local Contribution |
|---|---|---|
| DATA_0 | DATA_0 <= 0.00 | +0.0154 |
| DATA_5 | DATA_5 <= 0.00 | +0.0144 |
| DATA_1 | DATA_1 <= 0.00 | +0.0106 |
| DATA_7 | DATA_7 <= 0.00 | +0.0098 |
| DATA_6 | DATA_6 <= 0.00 | +0.0085 |
| DATA_3 | DATA_3 <= 0.00 | +0.0062 |
| DATA_4 | DATA_4 <= 0.00 | +0.0045 |
| DATA_2 | DATA_2 <= 0.00 | +0.0015 |

Notably, **all eight features** contributed positively to the final prediction, although to varying extents. No significant negatively contributing features were observed for this instance.

**2. Local Feature Prioritization Discussion**

The model's local prioritization indicates that the **lower byte values (≤ 0.00) across multiple fields** collectively pushed the instance toward a DoS classification. The ranking of feature contributions shows that **DATA_0** and **DATA_5** had the most substantial local impact, followed by **DATA_1** and **DATA_7**. This behavior suggests that specific patterns of **zero-valued bytes** across these fields were recognized by the model as characteristic signatures of DoS-type intrusions. The model appears to **aggregate weak but consistent signals** from multiple byte fields rather than relying on a single dominant feature for this decision.

**3. Interpretation of Features with Minimal or Near-Zero Contribution**

While all listed features contributed positively, the contribution from **DATA_2 (0.0015)** and **DATA_4 (0.0045)** was relatively low compared to others. This implies that although

the feature conditions for these bytes (DATA_2 <= 0.00, DATA_4 <= 0.00) were satisfied, their impact on the decision was marginal. Possible reasons include:

- **Low Discriminative Power:** These bytes might exhibit a zero value across both benign and malicious traffic, offering little unique information.
- **Feature Redundancy:** They could be correlated with stronger features (e.g., DATA_0, DATA_5), thus adding minimal incremental information.

**4. Logical Analysis of the Local Decision-Making Process**

The decision-making process revealed by LIME suggests that the model identified a **multi-byte zero-value pattern** as indicative of a DoS attack. In CAN traffic, DoS attacks often manifest as repetitive or malformed message patterns, potentially leading to zeroed-out payloads in certain bytes. The instance-level aggregation of multiple satisfied zero-value conditions points to a robust heuristic the model has developed to detect such anomalies:

- Rather than depending heavily on a single feature trigger, the model leverages **distributed weak signals** to achieve a moderate confidence (51%) in its prediction.
- The cumulative effect of several low-value contributions across the byte fields ultimately tipped the model's output toward the DoS class.

This behavior reflects a **distributed evidence accumulation strategy**, appropriate for detecting subtle anomalies in high-dimensional, noisy input spaces like CAN payloads.

**5. Alignment with Global Feature Importance Patterns**

Comparing the local importance observed here with the previously reported **global feature importance**:

| Feature | Global Importance Rank | Observed Local Contribution |
|---|---|---|
| DATA_6 | 1st | Moderate (+0.0085) |
| DATA_0 | 2nd | High (+0.0154) |
| DATA_1 | 3rd | Moderate (+0.0106) |
| DATA_5 | 5th | High (+0.0144) |
| DATA_3 | 4th | Lower (+0.0062) |
| DATA_4 | 6th | Minimal (+0.0045) |
| DATA_2 | 8th | Minimal (+0.0015) |
| DATA_7 | 9th | Moderate (+0.0098) |

Observations:

- The model's local decision largely aligns with global importance patterns, particularly emphasizing **DATA_0**, **DATA_5**, and **DATA_1**, consistent with their global prominence.
- However, **DATA_6**, despite being the most important feature globally, only contributed moderately here. This suggests that for this specific instance, the anomaly signal was **distributed across other byte fields**, and **DATA_6** alone was insufficient to drive the prediction.
- Lower-ranked global features such as **DATA_2** and **DATA_7** had minor yet nonzero local contributions, reinforcing that the model can flexibly adapt its feature reliance based on instance-specific evidence.

This divergence reveals that **LightGBM's decision boundary is highly instance-adaptive**, tailoring its reliance on features dynamically depending on the distribution of evidence, rather than applying a static feature weighting.

**Conclusion**

The LIME-based local interpretation demonstrates that the model, for this specific instance, classified the CAN message as a DoS attack by aggregating weak but consistent zero-value signals across multiple byte fields. The local feature usage partially mirrors the global importance ranking but also highlights the model's dynamic and in-

stance-sensitive decision-making behavior. This capacity for **adaptive feature prioritization** enhances both the robustness and interpretability of the CAN intrusion detection system, reinforcing its suitability for deployment in real-world automotive cybersecurity applications.

## B. Additional Results of Ablation Study

Based on the three specific instances introduced in Section A, this section presents additional results of the ablation study. The experimental findings indicate that the absence of knowledge derived from global feature importance, as obtained through the VLM, reduces the interpretability of the VLM-based LIME analysis. Specifically, when the global importance was excluded, the textual explanations generated by the VLM became less aligned with the model's actual decision boundaries, thereby lacking coherence. These results demonstrate the complementary role of global feature knowledge in enhancing the clarity and reliability of local explanation outputs, reinforcing the effectiveness of the proposed method in practical interpretability scenarios.

**Table S4.** Textual interpretation of LIME analysis except global feature importance using vision-language model – corresponding to Figure S1

**Result**

**Local Explanation for RPM-Classified CAN Message (LIME Analysis)**

**1. Summary of the Most Influential Features**

For this specific CAN message instance, the LightGBM-based CAN intrusion detection model classified the message into the **RPM** class with a **prediction probability of 1.00**, indicating complete confidence. According to the LIME analysis, the following feature conditions contributed positively toward the RPM classification:

| Feature Condition | Contribution (Local Weight) |
|---|---|
| DATA_3 > 137.00 | +0.00150 |
| DATA_7 > 109.00 | +0.00148 |
| 32.00 < DATA_5 ≤ 127.00 | +0.00144 |
| 8.00 < DATA_0 ≤ 69.00 | +0.00136 |
| 16.00 < DATA_4 ≤ 107.00 | +0.00128 |
| 32.00 < DATA_1 ≤ 69.00 | +0.00127 |
| 0.00 < DATA_2 ≤ 96.00 | +0.00117 |
| DATA_6 ≤ 0.00 | +0.00064 |

**Key Points:**

- All observed feature conditions contributed positively toward the RPM prediction.
- The highest individual positive influence comes from DATA_3 > 137.00 and DATA_7 > 109.00.
- No negatively contributing features were identified in this local explanation.

**2. Model's Local Feature Prioritization**

The model's decision-making emphasizes **high byte-level values** for DATA_3 and DATA_7 as the primary signals supporting the RPM classification:

- DATA_3 and DATA_7 both have extremely high actual values (255.00) compared to their threshold conditions (>137.00 and >109.00 respectively).
- These two features dominate the local explanation, suggesting that elevated values in specific CAN message bytes strongly signal RPM-related activities or anomalies.

Following these, mid-range values in DATA_5, DATA_0, DATA_4, and DATA_1 (all satisfying respective range conditions) further strengthen the classification decision, in-

dicating that **multiple mid-range byte checks** reinforce the prediction, not just outliers or maximum values.

DATA_2 and DATA_6 conditions also contribute positively, but with lower local weights, indicating secondary or supporting importance.

Overall, the model's local prioritization strategy here is a combination of:

- Recognizing **extreme high values** (DATA_3, DATA_7) as key indicators.
- Supplementing decision confidence through **validation of range-bound conditions** (DATA_5, DATA_0, DATA_4, DATA_1, DATA_2).

**3. Features with Minimal Contribution and Their Implications**

Among the listed features:

- DATA_6 ≤ 0.00 has the smallest contribution (+0.00064).
- Despite being satisfied (DATA_6 = 0.00 exactly), its relatively minor weight suggests that **absence or zero value in DATA_6 alone is not a strong predictor** of RPM behavior, but provides marginal supporting evidence.

Possible reasons for the minimal contribution:

- DATA_6 might commonly be zero or near-zero across multiple classes, reducing its discriminative power.
- Other features (such as DATA_3 and DATA_7) dominate the classification logic, rendering supporting conditions like DATA_6 less impactful.

No negative contributions (features pushing the prediction away from RPM) are observed, highlighting a highly consistent, aligned feature set for this specific decision.

**4. Byte-Level Local Decision Logic**

The LIME explanation reveals a **hierarchical decision logic** specific to this instance:

- The model first identifies **signature indicators** (DATA_3 > 137.00, DATA_7 > 109.00) to confirm strong alignment with RPM class characteristics.
- It then performs **secondary verifications** using range-based conditions across multiple other data bytes to consolidate the prediction.
- Minor contributions like DATA_6 ≤ 0.00 function as **weak supporting cues**, ensuring no contradictory signals are present.

This local decision process reflects a **multi-tiered byte-level pattern recognition** strategy:

- **Primary indicators**: Extreme values for specific bytes strongly hinting at RPM-specific message patterns.
- **Secondary confirmations**: Additional ranges validating consistency across the message.
- **Tertiary reassurances**: Weak features ensuring absence of contradictory evidence.

Such a layered approach is effective in CAN-based anomaly or event detection, where **specific bytes often encode critical operation states** like RPM changes.

**Conclusion**

The LIME analysis reveals that the model classified this instance into the RPM class by primarily leveraging extreme byte values in DATA_3 and DATA_7, with additional support from mid-range conditions in other features. Minor contributions, such as from DATA_6, acted as weak supporting cues. Overall, the model applied a clear and layered decision strategy, ensuring a confident and interpretable prediction for this CAN message.

**Table S5.** Textual interpretation of LIME analysis except global feature importance using vision-language model – corresponding to Figure S2

| Result |
|---|
| **LIME-Based Local Interpretation for CAN Intrusion Detection Instance (Classified as "Normal")** |

**1. Summary of Influential Features**

The following features exhibited the most significant impact on the model's local decision:

| Feature Condition | Contribution | Direction |
|---|---|---|
| DATA_6 ≤ 0.00 | +0.2725 | Positive toward "Normal" |
| DATA_2 > 96.00 | -0.2153 | Negative against "Normal" |
| DATA_5 ≤ 0.00 | +0.1693 | Positive toward "Normal" |
| DATA_7 ≤ 0.00 | +0.1617 | Positive toward "Normal" |
| DATA_0 ≤ 0.00 | +0.1167 | Positive toward "Normal" |
| DATA_4 ≤ 0.00 | +0.0979 | Positive toward "Normal" |
| DATA_1 ≤ 0.00 | +0.0621 | Positive toward "Normal" |
| 0.00 < DATA_3 ≤ 20.00 | +0.0505 | Positive toward "Normal" |

- **DATA_6 ≤ 0.00** emerged as the most critical positive driver, providing the largest contribution toward the prediction of the "Normal" class.
- **DATA_2 > 96.00** is the only feature with a strong negative influence, opposing the "Normal" classification.

**2. Feature Prioritization by the Model**

The LightGBM model prioritized features based on threshold-based split conditions at the byte level:

- **Dominant Positive Signals**: Features such as DATA_6, DATA_5, DATA_7, and DATA_0, when their byte values are at or below zero, strongly support the "Normal" prediction. This reflects a model behavior where the absence or minimal presence of certain byte patterns is characteristic of benign CAN traffic.
- **Negative Signal**: A notably large value in **DATA_2** (greater than 96.00) contradicted the "Normal" label, suggesting that higher byte values in this feature are often associated with non-normal (possibly attack) states.

Thus, the model relies on a **multi-condition aggregation**, predominantly favoring "normalcy" indicated by low or zero values in key byte positions, but is partially conflicted by the anomalous observation in DATA_2.

**3. Features with Minimal Contribution**

No features are shown with strictly negligible contributions in the visualized decision path; however:

- **DATA_3** exhibited the lowest positive contribution (+0.0505), suggesting limited relevance compared to other features.
- The relatively low weight assigned to **DATA_3** implies that minor variations in this byte range (0.00 < DATA_3 ≤ 20.00) have minimal impact on altering the prediction, possibly because such byte values are common across both normal and attack states.

The limited contribution could arise because:

- The observed value falls within a "neutral" or non-discriminative range.
- Other stronger signals (e.g., DATA_6 and DATA_5 conditions) overshadow the marginal effect of DATA_3.

**4. Logical Analysis of the Local Decision-Making Process**

The local decision boundary inferred by LIME suggests the model's decision is primarily driven by **absence or minimal activation** of certain bytes (DATA_0, DATA_1, DATA_4,

DATA_5, DATA_6, DATA_7), aligning with expectations for unperturbed, normal CAN communications.
However, the anomalously **high value in DATA_2** introduces uncertainty, pulling the prediction slightly away from "Normal," though not enough to overturn the decision. This tension illustrates how the model weighs cumulative evidence: **a majority of strongly positive conditions** can dominate **a single strong negative signal** if overall confidence remains high (as reflected in the 0.90 probability).
Given the byte-level nature of CAN messages, where each byte can encode discrete functional information, the model's reliance on specific byte thresholds demonstrates its effective learning of subtle protocol invariants distinguishing normal from abnormal states.
**Conclusion**
The LIME analysis shows that the model's classification of this instance as "Normal" is primarily driven by low or zero byte values across several key features, with a single conflicting signal from DATA_2. Despite the negative influence of DATA_2, the cumulative strength of multiple positive conditions secured a confident prediction. This layered evidence aggregation reflects the model's robust strategy for distinguishing benign CAN traffic based on characteristic byte-level patterns.

**Table S6.** Textual interpretation of LIME analysis except global feature importance using vision-language model – corresponding to Figure S3

**Result**

**LIME-Based Local Interpretation for CAN Intrusion Detection Instance (Classified as "DoS")**
**1. Summary of Influential Features**
The LIME analysis identifies the following feature conditions as the most influential for the model's classification of the instance into the "DoS" class with the highest probability (0.51):

| Feature Condition | Contribution | Direction |
|---|---|---|
| DATA_0 ≤ 0.00 | +0.0154 | Positive toward "DoS" |
| DATA_5 ≤ 0.00 | +0.0144 | Positive toward "DoS" |
| DATA_1 ≤ 0.00 | +0.0106 | Positive toward "DoS" |
| DATA_7 ≤ 0.00 | +0.0098 | Positive toward "DoS" |
| DATA_6 ≤ 0.00 | +0.0085 | Positive toward "DoS" |
| DATA_3 ≤ 0.00 | +0.0062 | Positive toward "DoS" |
| DATA_4 ≤ 0.00 | +0.0045 | Positive toward "DoS" |
| DATA_2 ≤ 0.00 | +0.0015 | Positive toward "DoS" |

Notably, all contributing feature conditions reinforce the prediction toward "DoS" (positive contributions). No features were identified as negatively impacting the "DoS" prediction for this instance.
**2. Model's Local Prioritization of Features**
The model predominantly relied on a sequence of features exhibiting values less than or equal to zero across multiple byte-level attributes (DATA_0 to DATA_7). The strongest contributors were:

- **DATA_0** and **DATA_5**, together accounting for the largest individual contributions, suggesting that specific byte inactivity or zero-value characteristics in these features are highly indicative of a DoS attack in this local context.
- **DATA_1** and **DATA_7** also play important supporting roles, reinforcing the anomaly pattern.

The cumulative effect of these features, even though individually modest, collectively

builds sufficient evidence for the model to confidently classify the instance as a DoS attack. This highlights the model's sensitivity to detecting systematic null or idle byte patterns that are characteristic of denial-of-service behaviors in CAN networks.

**3. Interpretation of Features with Minimal or Near-Zero Contribution**

- **DATA_2** (Contribution: +0.0015) and **DATA_4** (Contribution: +0.0045) exhibit the smallest positive contributions.
- These minimal contributions imply that while the feature conditions (DATA_2 ≤ 0.00 and DATA_4 ≤ 0.00) are consistent with the overall anomaly pattern, their standalone discriminative power for this particular prediction is limited.
- Possible reasons for their lower influence include:
  - **Redundancy**: These features may provide redundant information already captured by stronger features like DATA_0 and DATA_5.
  - **Lower Sensitivity**: The model may have learned that anomalies in these bytes are less consistently associated with DoS behavior compared to anomalies in other bytes.

**4. Logical Analysis of Local Decision-Making Process**

The local decision pathway, as exposed by LIME, suggests a **threshold-based binary assessment** where multiple byte values being at or below zero acted cumulatively to confirm an intrusion pattern typical of DoS attacks.

In the context of CAN message interpretation:

- A series of zeroed or inactive data bytes (DATA_0 through DATA_7 all being ≤ 0) may reflect **network inactivity or jamming patterns**, both of which are symptomatic of DoS conditions in vehicle networks.
- The LightGBM model appears to exploit this behavior, giving more weight to the absence of signal variability across multiple data fields.
- No negative contributions were identified, implying no contradictory signals were detected that could have challenged the DoS classification in this case.

Overall, the model’s local behavior is coherent with domain knowledge: in CAN intrusion detection, sustained null messages across critical bytes often indicate malicious flooding or bus-off attacks, both of which are associated with denial-of-service activities.

**Conclusion**

The LIME-based explanation shows that the model’s DoS classification is mainly driven by multiple zero-valued CAN data features, notably DATA_0 and DATA_5. The consistent positive contributions without any opposing features highlight the model’s confident decision-making. This behavior matches domain expectations, where DoS attacks are reflected by inactivity or uniformity across CAN messages. Overall, the analysis confirms the model’s robustness in detecting subtle byte-level anomalies.
